# Size and shape of plates and size of wine glasses and bottles: impact on self-serving of food and alcohol

**DOI:** 10.1186/s40359-021-00645-z

**Published:** 2021-10-20

**Authors:** Natasha Clarke, Emily Pechey, Rachel Pechey, Minna Ventsel, Eleni Mantzari, Katie De-loyde, Mark A. Pilling, Richard W. Morris, Theresa M. Marteau, Gareth J. Hollands

**Affiliations:** 1 Behaviour and Health Research Unit, Department of Public Health and Primary Care, University of Cambridge, Cambridge, UK; 2 Nuffield Department of Primary Care Health Sciences, University of Oxford, Oxford, UK; 3 Tobacco and Alcohol Research Group, School of Psychological Science, University of Bristol, Bristol, UK; 4 Bristol Medical School, University of Bristol, Bristol, UK

**Keywords:** Size intervention, Plates, Wine glasses, Wine bottles, Alcohol, Food

## Abstract

**Background:**

The physical properties of tableware could influence selection and consumption of food and alcohol. There is considerable uncertainty, however, around the potential effects of different sizes and shapes of tableware on how much food and alcohol people self-serve. These studies aimed to estimate the impact of: 1. Plate size and shape on amount of food self-served; 2.Wine glass and bottle size on amount of wine self-poured.

**Methods:**

140 adults participated in two laboratory studies—each using randomised within-subjects factorial designs—where they self-served food (Study 1) and wine (Study 2):

Study 1: 3 plate sizes (small; medium; large) × 2 plate shapes (circular; square).

Study 2: 3 wine glass sizes (small; medium; large) × 2 wine bottle sizes (75 cl; 50 cl).

**Results:**

Study 1: There was a main effect of plate size: less was self-served on small (76 g less, *p* < 0.001) and medium (41 g less, *p* < 0.001) plates, compared to large plates. There was no evidence for a main effect of plate shape (*p* = 0.46) or a size and shape interaction (*p* = 0.47).

Study 2: There was a main effect of glass size: less was self-served in small (34 ml less, *p* < 0.001) and medium (17 ml less, *p* < 0.001) glasses, compared to large glasses. There was no evidence of a main effect of bottle size (*p* = 0.20) or a glass and bottle size interaction (*p* = 0.18).

**Conclusions:**

Smaller tableware (i.e. plates and wine glasses) decreases the amount of food and wine self-served in an initial serving. Future studies are required to generate estimates on selection and consumption in real world settings when numerous servings are possible.

**Protocol registration information**: OSF (https://osf.io/dj3c6/) and ISRCTN (10.1186/ISRCTN66774780).

**Supplementary Information:**

The online version contains supplementary material available at 10.1186/s40359-021-00645-z.

## Introduction

Excess consumption of alcohol and energy-dense foods are two significant preventable causes of a range of non-communicable diseases globally, including heart disease and many cancers [1–4]. Interventions that involve changing cues in the immediate physical environments that influence consumption could contribute to reducing excess food and alcohol consumption [5]. Altering the size of tableware, including plates and glasses, and packaging, including bottles, is one intervention that has received considerable attention.

Evidence is inconclusive as to whether the size of tableware impacts on the amount of food consumed. One systematic review found no consistent effect of larger tableware on consumption [6], a Cochrane review found a small to medium effect [7], while the most recent meta-analysis [8] found a substantial effect. These meta-analyses specified different inclusion criteria for the consumption outcomes which may contribute to the observed differences in effect sizes. Importantly, in Holden et al. [8], the overall effect was explained by a large effect of plate size on consumption when food was self-served, with a minimal effect when portion size was held constant, i.e. when people were given pre-served portions. Given selection (including serving on to a plate) of food is a necessary precursor to consumption, some studies have examined this outcome separately to consumption, with the aforementioned Cochrane review by Hollands et al. [7] finding a medium-sized effect of plate size on selection. However, most studies included in previous reviews have used small samples, and been at substantial risk of bias. Notably, for the selection outcome in the Cochrane review meta-analysis, of seven comparisons included, the majority (four) were authored by an individual who has been subject to multiple retractions of their work due to academic misconduct [9, 10], meaning that the veracity of these data should be regarded with caution. Furthermore, current guidance recommends that research workflows are transparent and open [9], but none of these previous studies were pre-registered.

We are only aware of one pre-registered study that has applied rigorous randomised controlled trial procedures to examine the effects of tableware on selection and consumption [11]. This study found no clear evidence of a statistically significant difference in either the total amount selected or consumed over a whole meal period when food was self-served on to a smaller (23 cm diameter) compared to a larger (29 cm diameter) round plate. However, the possibility of an important effect—in either direction—could not be excluded, as even small reductions in consumption (in this case equivalent to a 3% difference) could be meaningful in terms of public health impact. Due to varying effect size estimates observed in previous studies, uncertainty remains around potential effects. The aforementioned study [11] compared only two plate sizes, while previous studies have compared a wide variety of sizes. For example, in one meta-analysis, plates sizes ranged from 17 to 27.5 cm [6]. Further robust studies are warranted to investigate differences in selection and consumption with a larger range of plate sizes. The mechanisms for any potential effects of plate size remain unclear [12], but include perceptual effects—where perceived portion size becomes distorted by the size of the dish on which it is served, i.e. as in the Delboeuf illusion [13]—and the maximum capacity of the plate.

In terms of alcohol, current evidence from a small number of studies suggests that the size of tableware—such as glasses—can influence drinking behaviour in field settings. For example, a recent mega-analysis [14] of data from eight pre-registered studies conducted in five establishments found that larger wine glasses increased the volume of wine sold—a proxy for consumption—in restaurants, but not in bars. This may reflect greater sales in restaurants of bottles and carafes requiring free-pouring of wine. Nearly all the wine sold in bars included in this mega-analysis was by the glass—i.e. containing a fixed amount of wine served by bar staff—suggesting that glass size has less impact on the amount consumed when wine is sold in fixed servings. Pouring into larger containers can increase the amount of beer and spirits poured [15], but this has yet to be investigated with pouring wine into wine glasses, and in particular in the glass sizes used in recent field studies [14].

In addition to size, the shape of tableware also has the potential to influence consumption. For example, the shape of glasses can influence consumption of alcoholic and non-alcoholic drinks. Outwardly curved glasses can increase the rate of consumption of alcohol [16] and soft drinks [17, 18] compared to straight-sided glasses. There is an absence of direct evidence, however, concerning the impact of tableware shape upon food selection and consumption; the aforementioned Cochrane review [7] did not identify any such studies. There is some preliminary evidence that circular compared with square shaped plates might influence cognitive processes related to consumption, with one study finding food eaten from circular plates was more highly rated in taste, quality and liking [19]. This study, however, used a small sample, and surface area was not kept constant between the circular and square plates, thus generating uncertainty over the reliability of the findings. Another study with hospital patients found that ratings of flavour and taste, appearance and food quality were higher for oblong or rectangular shaped plates compared to circular shaped plates [20].

The size of the packaging in which products are presented and sold might also influence consumption, with larger packages found to increase consumption of food [7]. Bottle size may also influence wine consumption. A recent randomised controlled trial [21] found that consuming wine at home from 50 cl bottles decreased the amount consumed by 4.5% and slowed the rate of consumption by almost 6% compared to consumption from 75 cl bottles. The mechanisms by which smaller bottles may reduce wine consumption are unknown. For example, it is unclear whether consumption is reduced because individuals pour less from smaller bottles. Given the amount poured and thus consumed may also be influenced by glass size, it is also important to examine the possible interaction between bottle and wine glass size.

The current research, which consists of two laboratory studies, focuses on selection—self-serving and self-pouring—of food and wine, a reliable, proximal determinant of consumption. In contexts where people self-serve food and drink to then consume, consumption is dependent on, and has been shown in some studies to be near-identical in value to, selection. For example, studies demonstrate correlations of r = 0.98–0.99 between amount served and amount consumed [11, 22] and in alcohol serving size studies most participants consume the drinks they purchase—with less than 1% waste reported (email communication with Inge Kersbergen, PhD, 2019 [23]).

This paper presents two laboratory studies that aimed to estimate the impact of plate size and shape on the amount of food self-served (Study 1), and the impact of size of wine glasses and wine bottles on the amount of wine self-poured (Study 2).

### Hypotheses

#### Study 1

(i) As plates increase in size, an increasing amount of food is self-served onto them.

There was insufficient evidence for a directional hypothesis for plate shape.

#### Study 2

(i) As wine glasses increase in size, an increasing amount of wine is self-poured.
(ii) More wine is poured from larger bottles compared to smaller bottles.
(iii) The effect of increasing bottle size is more marked for larger glasses than for smaller glasses.

## Methods

These two studies were preregistered in one protocol prior to data collection on OSF (https://osf.io/dj3c6/) and ISRCTN (10.1186/ISRCTN66774780).

### Design

Two randomised within-subjects factorial studies, each with six conditions. All participants completed both Study 1 and Study 2 sequentially, with the order of studies being randomised.

*Study 1: Plate size and shape.* 3 plate sizes (small, medium, large) × 2 plate shapes (circular; square). Participants were randomised to the order of plates onto which they self-served food, determined by simple randomisation.

*Study 2: Glass and bottle size.* 3 wine glass sizes (29 cl, 35 cl, 45 cl) × 2 wine bottle sizes (75 cl; 50 cl). Participants were randomised to the order in which they self-poured from each of the bottles into each of the glasses, determined by block randomisation.

Separate randomisations were produced by computer generated random sequences with sealed envelope [24] by a statistician not involved with data collection, to determine (a) the order in which participants completed Study 1 and 2, (b) the order in which plates were presented during Study 1, (c) the order in which the combination of wine glasses and bottles were presented during Study 2. The statistician completing the randomisation was blinded to allocation.

According to the Typology of Interventions in Proximal Physical Micro-Environments (TIPPME) [5] these manipulations are categorised as *Product × Size* interventions.

### Participants

Participants were 140 adults from the general population, recruited via a research agency.

To be eligible to take part, participants had to eat rice and drink white wine at least once a month, be over the age of 18, and be able to read and write in English.

#### Sample size

It was originally planned to recruit a sample of 279 participants. To detect a small effect (d = 0.2) with power = 0.90, alpha = 0.05 and a conservative estimate of 0.5 for within person correlations [25], 265 participants were needed. Based on the previous plate size study [11], in which randomisation occurred immediately prior to a single study session, a dropout rate of no more than 5% was anticipated (see pre-registered protocol for more details: https://osf.io/dj3c6/).

This research was terminated prematurely on 16/03/2020, due to the Covid-19 pandemic. Analyses were therefore conducted on data from the number of participants who had already completed the studies by that date (n = 140). It is estimated that this final sample size gives a power of 65% to detect the anticipated small effect size (d = 0.2) or a power of 80% to detect d = 0.276.

### Materials (see Fig. 1a, b)

**Fig. 1:**
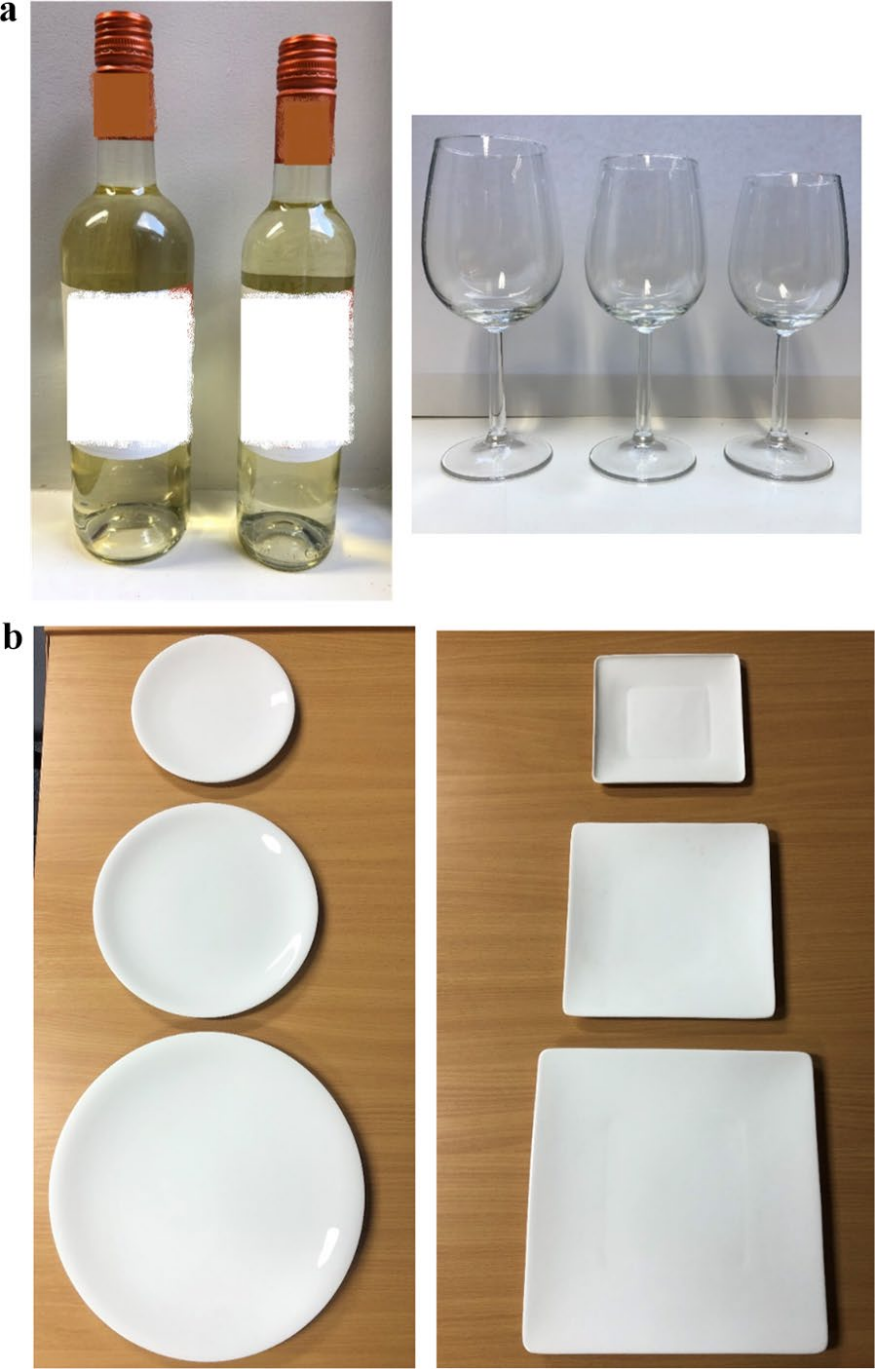
**a** Wine bottles (75 cl & 50 cl) and glasses (45 cl, 35 cl & 29 cl). **b** Round (diameter: 18 cm, 23 cm & 29.5 cm) and square (side length: 16 cm, 20.4 cm & 26.1 cm) plates

#### Plates (Study 1)

Three different sized (small, medium, large) and two different shaped (circular, square) plates were used in the study, varying in surface areas: (i) Small: 254.5 cm^2^ (18 cm diameter circular plate; 16 cm square plate); (ii) Medium: 415.5 cm^2^ (23 cm diameter circular; 20.4 cm square); (iii) Large: 683.5 cm^2^ (29.5 cm diameter circular; 26.1 cm square). The surface areas were kept constant (within 3%) between different shaped plates of the same size. The set of sizes was informed by the sizes of circular plates previously used in a laboratory study [11] (23 and 29 cm).

All plates used were of a similar design, made from white bone china, unlined and unpatterned—with custom-made plates made as necessary (Circular plates: China by Denby Dinner Plate [large], China by Denby Dessert/Salad Plate [medium], China by Denby Tea Plate [small]; Square plates: Bennett Square Dinner Plate [large], Bennett Square Plate Salad Plate [medium], Custom Made by Reiko Kaneko [small]).

#### Glasses and bottles (Study 2)

Three sizes of wine glasses were used in the study with capacities of: (i) 29 cl (small), (ii) 35 cl (medium), (iii) 45 cl (large). This set of sizes was informed by those used in recent field studies (Clarke et al., 2019; Pilling et al., 2020). All glasses were of the same design (Royal Leerdam Bouquet).

Two wine bottle sizes were used in the study: (i) 50 cl, (ii) 75 cl. The set of bottle sizes was informed by a recent randomised controlled trial assessing the impact of bottle size on wine consumption [21]. Both bottles contained the same wine, using a branded bottle to increase ecological validity (Isla Negra Sauvignon Blanc: 13.2% alcohol by volume (abv)). The grape variety was chosen on the basis of it being the most popular in the UK. The brand was chosen based on it being available in both 50 cl and 75 cl sizes and in bottles of identical design, i.e. shape and colour.

### Setting

Laboratory setting, conducted in a general-purpose function room of St Andrew’s Street Baptist Church, Cambridge.

### Measures

#### Primary outcomes

##### Study 1: plate size and shape

Amount of food (in grams) self-served.

##### Study 2: glass and bottle size

Amount of wine (in millilitres) self-poured.

#### Additional measures

##### Demographics

Self-reported age, sex, ethnicity, height, weight and education (highest qualification).

##### Filler questions

Asked after each serve/pour to reinforce the cover story (i.e. that this study was investigating the visual appeal of food and wine presented on/in different tableware/ glassware). Filler questions included, Study 1: (a) How visually appealing do you think this food is on this plate? (b) Would you like to eat/drink from this plate/glass?; Study 2: (a) How easy was it to pour from this bottle into this glass? (b) How visually appealing do you think the wine looks in this glass? Each question was answered via a 7-point scale (0 = Not at all; 7 = Very).

##### Perceived aims of the study

Assessed to measure effectiveness of the cover story. The free-text question asked: ‘Please use the space below to briefly tell us what you think the study was about’.

### Procedure

Ethical approval was granted by the Cambridge Psychology Research Ethics Committee (PRE.2019.097).

Participants provided written informed consent to participate in both studies that formed part of this research and were told they could withdraw from the study sessions at any time. They were given a cover story that the research was examining how different plates and glasses affect the visual appeal and attractiveness of food and drink. To enhance the cover story, participants were first presented with a range of tableware and glassware of different colours, shapes and sizes, and asked to select their preferred options and explain why they found these designs visually appealing (not used in the analysis). They then completed Study 1 and Study 2 in a randomised order, with one immediately following the other (see Additional file 1: S1 for details on a difference from the pre-registered protocol).

For Study 1, participants were randomised to the order of the six plate conditions. Participants were first presented with a short context-setting vignette (i.e. “Imagine the most typical setting in which you serve yourself a meal. Please serve your typical amount”) and for each plate condition they were asked to self-serve their typical amount of food from a serving dish containing more than could be feasibly served on to the largest plate (1.5 kg of Uncle Ben’s Golden Vegetable Rice [156 calories per 100 g]). Participants were informed that they were not required to consume the food. To minimise any potential effects of awareness of being observed, the researcher was not present when participants were self-serving as they left the room during each serving period. Each portion of self-served food was weighed (by weighing the remaining food in the serving bowl) and the serving bowl was re-filled between serving sessions, to ensure the amount of food remained constant across conditions. The researcher weighed and re-filled the serving bowl in another room while participants completed filler questions in line with the cover story, and additional distraction tasks (e.g. word searches and drawing activities). Each of the six plates were presented separately, with all other plates kept hidden during each self-serving session.

For Study 2, participants were randomised to the order of the six wine bottle × glass conditions. Participants were first presented with a short context-setting vignette (i.e. “Imagine the most typical occasion in which you pour yourself wine. Please pour your typical amount”) and for each glass/bottle condition they were asked to self-serve their typical amount from the provided bottle (Isla Negra Sauvignon Blanc: 13.2% abv). Participants were informed they were not required to consume the wine. The remaining procedure is identical to that for Study 1.

After completing both studies, participants completed the demographic measures (age, sex, education, ethnicity, height and weight), then answered questions on what they believed the study was about, were debriefed, and reimbursed 30GBP for their time.

### Data analysis plan

The detailed data analysis plan was pre-registered prior to analysis (https://osf.io/dj3c6/).

For both studies the primary analysis consisted of a 3 × 2 repeated measures general linear mixed model. The initial models included the two main effects and the 2-way interactions. In line with the analysis plan, the interactions were removed from both models as the *p* value was > 0.01, therefore the final models include the main effects only. This was visualised using marginal effect plots [26].

Free-text comments for the aim of the study question were coded by one researcher, with another researcher independently coding 10% of responses.

For sensitivity analyses, the primary analysis for both studies were repeated, excluding participants that guessed the true nature of the study, those identified by the researcher as incorrectly following instructions and excluding extreme outliers.

Primary analysis was also repeated including covariates for study order (whether Study 1 or Study 2 came first), the order in which self-servings occurred and unbalanced variables (gender and education).

To further address the research hypothesis, two further mixed models were fitted where plate area and glass volume were used as a continuous covariate to assess the linear increase in the amount self-served, which was appropriate given the linearity of effects.

All model residuals were checked and were seen in normal plots not to markedly depart from a Normal distribution.

## Results

### Participant characteristics

**Fig. 2:**
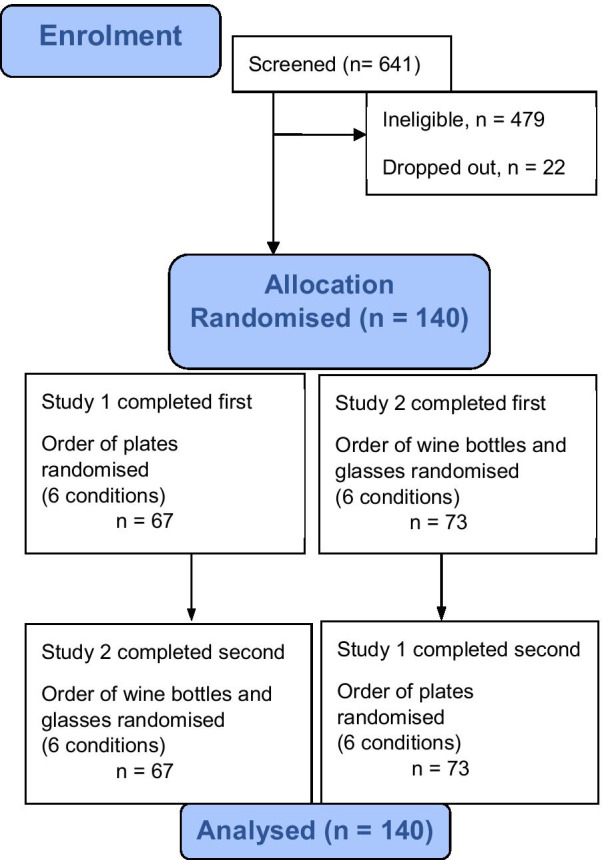
Study flow diagram

In total, the same 140 participants completed both studies (Fig. 2). Study order and the order of conditions were well balanced. Participant characteristics are presented in Table 1. The mean age of the sample was 40.7 (SD = 14.1) and 69% were female (n = 96).

**Table 1:** Participant characteristics

Demographic characteristic	n*	%*
Sex		
Male	44	31
Female	96	69
Age, mean (SD)	40.7 (14.1), range 18–71	
Ethnicity		
White–British	115	82
Non-white	25	18
Education (highest qualification)		
No qualifications	1	< 1
Up to 4 GCSEs	10	7
5 or more GCSEs or 1 A-level	16	12
2 or more A-levels	30	22
Bachelor’s degree	40	29
Post-Graduate degree or qualification	42	30
Missing	1	
BMI (grouped)		
Underweight (under 18.5)	4	3
Healthy weight (18.5–24.9)	66	51
Overweight (25–29.9)	36	28
Obese (30–34.9)	18	14
Severely obese (35–39.9)	2	2
Morbidly obese (40+)	3	2
Prefer not to say	5	
Missing	6	
BMI, mean (SD)	25.6 (6.6), range 16–65	
Study completed first		
Study 1	67	48
Study 2	73	52

*unless otherwise stated. Note: Missing/prefer not to answer data is listed in the table but all % are valid%

BMI: body mass index; SD: standard deviation

### Primary outcomes

*Study 1*.

**Fig. 3:**
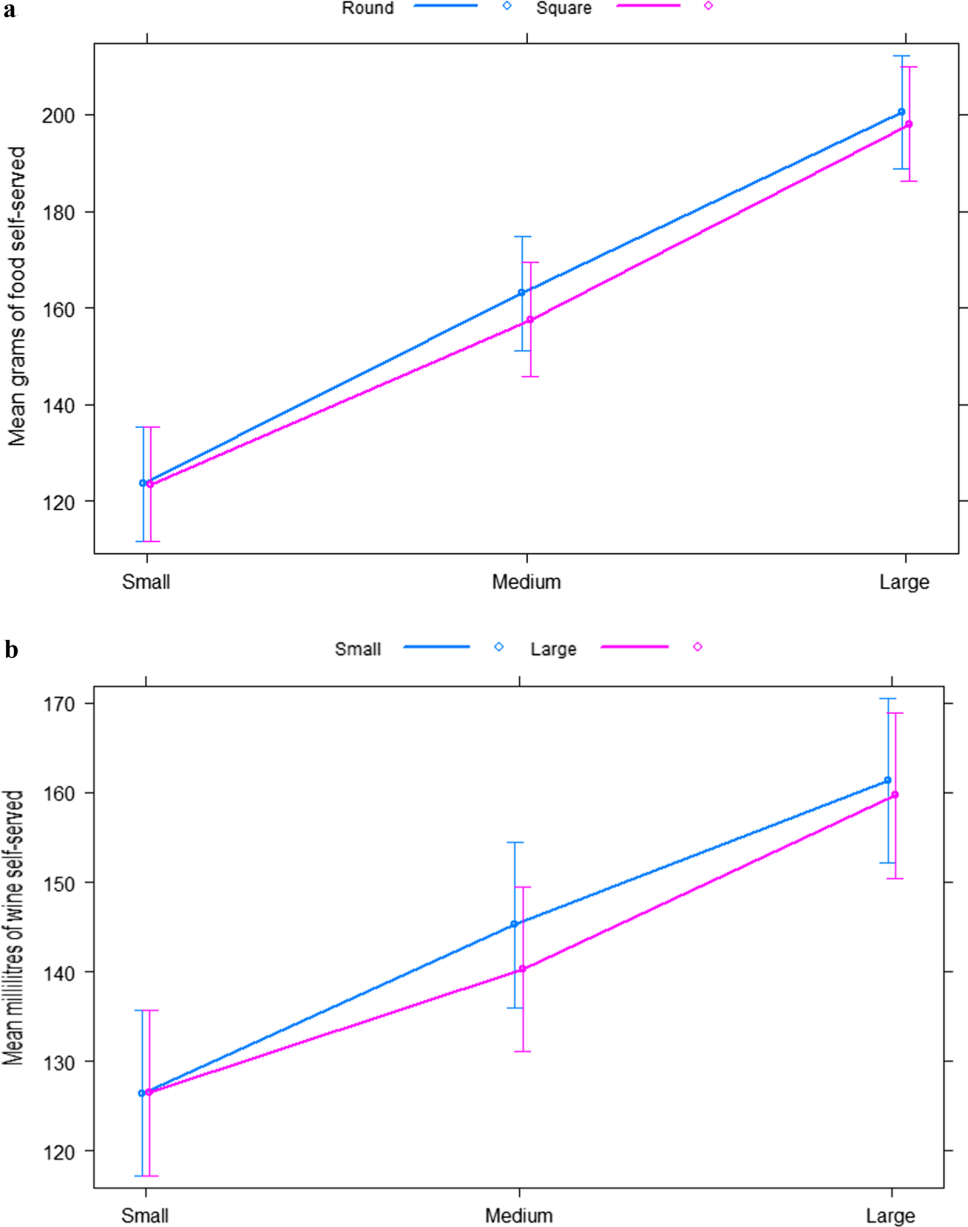
**a** Marginal effects plots of the relationship between plate size and plate shape. Error bars are 95% CIs. **b** Marginal effects plots of the relationship between glass size and bottle size. Error bars are 95% CIs

Raw means are presented in Table 2; Fig. 3a.

**Table 2:** Primary outcomes. Raw means

Study 1: Plate size and shape	Grams of food self-served	
	Mean	SD
Square		
Small plate	123.5	48.4
Medium plate	157.5	62.9
Large plate	198.1	97.8
Round		
Small plate	123.5	50.0
Medium plate	163.0	69.6
Large plate	200.5	83.9

Study 2: Wine glass and bottle size	Millilitres of wine self-served	
	Mean	SD
Small bottle		
Small glass	126.4	46.8
Medium glass	145.3	52.7
Large glass	161.4	62.2
Large bottle		
Small glass	126.5	46.8
Medium glass	140.3	54.9
Large glass	159.7	67.4

In a repeated-measures 3 (plate size: small vs. medium vs. large) × 2 (plate shape: square vs. circular) model, there was evidence of an overall main effect of plate size (*p* < 0.001), no evidence of a main effect of plate shape (*p* = 0.46) and no evidence of an interaction between plate size and plate shape (*p* = 0.47).

For the main effect of plate size, compared to the large plate, there was a reduction in the amount self-served in the small plate (− 76.4 g [95% CI − 86.7, − 66.2], t (139) = − 14.81, *p* < 0.001, d = − 1.54) and the medium plate (− 40.5 g [95% CI − 48.2, − 32.9], t (139) = − 10.49, *p* < 0.001, d = − 0.79) conditions (see Table 3). Participants served a mean amount of 123.5 g (equivalent to 192.6 kcal) in the small plate condition, 160.3 g (250 kcal) in the medium plate condition and 199.3 g (310.9 kcal) in the large plate condition (raw means). There was an increase of 0.17 g (95% CI 0.15 to 0.20) with each 1cm^2^ increase in plate size.

**Table 3:** Estimated mean differences and model effects for primary outcomes

Study 1: Plate size and shape	Grams of food self-served			*p*	Cohen‘s *d* (95% CI)
	Estimated mean (SE)	Estimated MD	(95% CI of the MD)		
Plate size					
Small	123.6 (4.0)	− 76.4	− 86.7, − 66.2	< 0.001	− 1.5 (− 1.7, − 1.3)
Medium	159.6 (5.2)	− 40.5	− 48.2, − 32.9	< 0.001	− 0.8 (− 0.9, − 0.7)
Large	200.1 (6.8)	ref	–	–	–
Plate shape					
Square	160.3 (4.9)	− 1.5	− 5.6, 2.5	0.456	− 0.07 (− 0.21, 0.08)
Round	161.9 (5.1)	ref	–	–	–

Study 2: Wine glass and bottle size	Millilitres of wine self-served			***p***	Cohen‘s d (95% CI)
	Estimated mean (SE)	Estimated MD	(95% CI of the MD)		
Glass size					
Small	126.7 (3.8)	− 33.9	− 39.3, − 28.6	< 0.001	− 1.4 (− 1.5, − 1.2)
Medium	143.3 (4.35)	− 17.3	− 21.6, − 13.1	< 0.001	− 0.7 (− 0.9, − 0.6)
Large	160.6 (5.2)	ref	–	–	–
Bottle size					
Small	144.5 (4.3)	1.9	− 1.0, 4.8	0.200	0.11 (− 0.04, 0.26)
Large	142.6 (4.4)	ref	–	–	–

MD (Mean difference); SE (Standard error); CI (confidence interval)

*Study 2*.

Raw means are presented in Table 2; Fig. 3b.

In a repeated-measures 3 (wine glass size: small vs. medium vs. large) × 2 (wine bottle size: 50 cl vs. 75 cl) model, there was evidence of an overall main effect of wine glass size (*p* < 0.001), no evidence of a main effect of wine bottle size (*p* = 0.20) and no evidence of an interaction between wine glass size and wine bottle size (*p* = 0.18).

For the main effect of wine glass size, compared to the large glass, there was a reduction in the amount self-poured in the small glass (− 33.9ml [95 %CI − 39.3, − 28.6], t (139) = − 12.52, *p* < 0.001, d = − 1.40) and the medium glass (− 17.3ml [95 %CI − 21.6, − 13.1], t (139) = − 8.14, *p* < 0.001, d = − 0.72) conditions (see Table 3). Participants poured a mean amount of 126.5 ml (equivalent to 1.7 alcohol units) in the small glass condition, 142.8 ml (1.9 alcohol units) in the medium glass condition and 160.5 ml (2.1 alcohol units) in the large glass condition (raw means). There was an increase of 2.17 ml (95% CI 1.84, 2.50) served with each 1 cl increase in glass volume.

### Sensitivity analyses

Three sensitivity analyses were conducted for the analysis of the primary outcome for both studies. These analyses excluded participants who: (i) guessed the true nature of the study (n = 72) (Additional file 1: Table S1) and (ii) followed instructions incorrectly (n = 28) (Additional file 1: Table S2). The third sensitivity analysis (iii), removed an outlier participant who selected 893 g with the square large plate (Additional file 1: Table S3), which was 9 standard deviations higher than the mean. Conclusions were unchanged for all sensitivity analyses.

### Further analyses

When either the study order or the order in which self-servings occurred were added as a covariate, there was no evidence either of these had an effect (Additional file 1: Tables S4 and S5). Results also suggested no effect of the unbalanced variables (gender and highest education level) when including them as covariates.

Given such information may be useful for researchers planning similar studies, within-subjects correlations for the primary outcome were derived from mixed effects models. These were 0.62 for Study 1 and 0.84 for Study 2.

## Discussion

In a laboratory setting, larger plates increased the amount of food self-served and larger wine glasses increased the amount of wine self-poured, supporting two of the hypotheses tested. There was no evidence of a difference in amount of food served with different shaped plates and no evidence of a difference in the amount of wine poured with different sized wine bottles. The latter was contrary to the hypothesis under investigation. No directional hypothesis was formulated for the effect of plate shape.

In terms of plate size, the finding that larger plates led to larger servings—suggesting approximately linear differences between the three plate sizes—is in line with previous meta-analyses that show moderate to large effects of plate size on selection and consumption [7, 8]. However, the effect size lies outside the confidence intervals reported in another meta-analysis [6], as well as our previous study in a naturalistic eating laboratory [11], both of which showed no clear effect on selection or consumption. Previous studies report a range of effect sizes (*d* = 0.11 to 0.7) accompanied by wide confidence intervals, likely due to them being heterogeneous in their design (e.g. varying plate sizes, different procedures, range of outcomes) and often of poor quality design. The current study suggests there may be a large effect of plate size, at least on an initial serving of food. Given the lack of effect on selection and consumption over an extended mealtime observed in Kosīte et al. [11], in which numerous servings were possible, one explanation for this apparent inconsistency is that any effect of plate size may reduce when participants are consuming what they serve and are able to re-serve themselves repeatedly. While we are unaware of any direct evidence on the amount selected in a first serving when multiple servings are possible, in one previous study participants did serve more with a larger plate when only one trip to the buffet was allowed [27]. In another study, significantly more trips were made to the buffet with smaller plates [28]. Smaller plates may therefore lead to a smaller initial serving, and this may lead to reduced consumption but only if one serving is possible. Differences in initial servings may be in part due to the maximum capacity of the plates—with the large plate closest in size to that typically used in the real-world. Consistent with this, some participants of the current studies reported that they would have indeed served themselves more on small plates had it been possible. Another possibility is that differences are due to perceptual effects of plate size on perceived portion size [12, 13]. Further studies are required to estimate the impact of smaller plates when multiple servings are made, as well as to elucidate these potential mechanisms. Other factors, such as type of food or conscious engagement (i.e. focus on the serving task), may also influence the effect of plate size on serving, meriting further investigation. The degree of influence these factors might have could depend on the mechanism for the effect. For example, if the primary mechanism is through perceived perceptual effects of plate size on perceived portion size, the effect might be modified by the degree of conscious engagement (e.g. see Zlatevska et al. [29]). However, if effects are largely determined by the maximum capacity of the plate and therefore relate to a physical restriction, there may be less variation with changes in degree of conscious engagement. A deepened understanding of the mechanisms underlying the effect of plate size on serving, such as how this may be modified by the degree of conscious engagement of the individual [30], could have important implications for implementing and optimising any related interventions to change behaviour [31].

There was insufficient evidence at the study outset to specify a directional hypothesis for the effect of plate shape—square versus circular—on the amount of food self-served [7],  with existing evidence relating only to the impact of shape on perceptions of taste and quality of food, rather than selection or consumption [19, 20]. The current study provides the first experimental evidence of the effect of plate shape on selection, suggesting there is either no relationship or a very small effect that the current study was not powered to detect, even had the larger intended sample size been used.

In terms of wine glass size, the finding that larger glasses lead to larger amounts being self-poured is in line with a mega-analysis of field studies that showed larger glasses increased wine sales in restaurants, where wine is more likely to be free-poured by customers or staff [14]. This effect was found when comparing medium (370ml) to smaller (300 ml) glasses, but not with larger (450 ml) compared to smaller (300 ml) glasses. The current study, by contrast, suggested a linear difference in amount poured between all three glass sizes. If the relationship between glass size and amount poured is linear, it may be that other factors, such as compensatory behaviour across multiple drinks, may act in field but not laboratory settings, to limit the impact of differences in pouring. Other evidence from laboratory settings also report that participants over-fill larger glasses when attempting to match a reference glass [32] or pouring a standard drink [15]. There is, however, a near absence of evidence regarding impact on consumption. While current evidence is consistent with the suggestion that the effect observed in restaurants—that serving wine in larger glasses increases sales—is due to people self-pouring more wine into larger glasses, this awaits direct testing in studies that measure both self-pouring and consumption.

Finally, there was no effect of bottle size on pouring behaviour, which was not in line with the study hypothesis that larger bottles increase the volume poured. This hypothesis was, however, based on just one previous study [21] which found that wine consumption was higher from 75 cl bottles, compared to 50 cl bottles. The mechanisms for this effect are unknown but one possibility is that it may reflect the tendency for people to consume in ‘units’ [33], with one bottle—regardless of size—comprising one unit. In keeping with this explanation, when consuming wine from a bottle, a drinking episode—e.g. a meal or an evening—would be considered complete when the bottle is empty. The current study design could not test this mechanism but tested an additional possible explanation: less is poured from smaller bottles leading to overall lower consumption from such bottles. Studies are needed that assess both pouring and consumption when wine is consumed from bottles of different sizes.

### Strengths and limitations

These laboratory studies are the first to our knowledge to investigate the impact of plate size and shape on initial self-servings of food, and the impact of wine glass size and bottle size on amount of wine self-poured. The protocol and statistical analysis were pre-registered and the study procedures complied with randomised controlled trial guidance as far as possible, including rigorous randomisation procedures and analysis conducted by a statistician unaware of condition allocation. However, there are some limitations.

First, only half the pre-specified sample was recruited because the studies were halted due to the Covid-19 pandemic. The study was designed to recruit a balanced sample, with similar proportions of men and women and similar proportions within a low socioeconomic position (SEP) and within a high SEP. However, as the study was halted early, certain participant quotas were filled up more quickly, resulting in a sample that was disproportionately female (69%) and of higher SEP (80%) than intended. While this has possible implications for the generalisability of the study, further analyses showed no effect of these imbalanced covariates. Furthermore, the smaller than intended sample size reduced power. However, the statistically significant effect sizes of plate size and wine glass size were larger than predicted (d = 0.7–1.5), and any effects of plate shape and bottle size were likely to have been too small to detect (d = 0.07–0.11) even if the planned sample size had been collected.

Second, the studies measured serving behaviour rather than consumption. Although previous studies suggest selection is a close proxy for consumption as the two outcomes are very highly correlated [11, 22, 23], comparable effects on consumption cannot be assumed. Both of the current studies used a repeated measures design with six conditions, where it would have been impossible to measure consumption given finite physiological capacity for consumption of food and ethical considerations regarding alcohol intake. Furthermore, measuring consumption would have presented substantial challenges to completing the studies as taking significantly longer to conduct each condition would mean such a design would not be practicable to conduct. Nevertheless, future study designs with a narrower focus but that incorporate both consumption and serving measures are warranted.

Finally, the context had lower external validity than naturalistic eating laboratories and field settings, used in some previous studies assessing tableware interventions (e.g. Kosīte et al. [11]). However, the artificial laboratory setting provided controlled conditions for precise weighing of serving sizes, and the repeated measures design reduced potential error arising from variance in individuals’ typical serving size. This design also minimised the potential influence of differences between participants, such as in hunger status, as well as other possible confounding factors, such as time of day. Relatedly, many participants guessed the aim of the study despite our cover story and attempts to minimise participant awareness by displaying different tableware at the start of the study. This may be due to the fact that participants were completing multiple conditions in which only the size or shape of tableware changed. It should be noted, however, that the findings remained the same when excluding participants who had guessed the study aim. We consider it unlikely that awareness of the aim could have been addressed by changing the cover story and instead is likely linked to the challenges inherent in using a within-subjects design in this context, particularly as participants were exposed to a large number of conditions in succession. In addition, although attempts were made to minimise any effects of being observed by having the researcher leave during servings, the presence of the researcher may nonetheless have influenced selection. For example, participants eat less of an energy-dense snack under conditions of heightened awareness [34]. For these reasons, although the within-subjects design provides some important advantages, future studies may benefit from being conducted using between subjects designs in laboratory and naturalistic settings.

### Future research directions

Current findings suggest that there is an effect of tableware size on food and wine servings, with smaller tableware—i.e. plates and glasses—decreasing the amount initially self-served and self-poured. Previous research suggests that there are no effects of plate size on consumption when portion sizes are held constant [8]. Studies are therefore warranted that assess initial as well as subsequent servings, and consumption behaviour—including detailed meal microstructure behaviours—over time in naturalistic laboratory and field settings. It should be acknowledged, however, that identifying and gaining access to such naturalistic settings can prove challenging, particularly given commercial interests are often involved. Another specific challenge is gaining access to field settings where changing plate sizes is feasible as an intervention. The current study and previous literature suggest that it is when participants self-serve their food where effects are more clearly observed [29]. This suggests that plate size is a difficult intervention to test in field settings, particularly in restaurants or cafeterias where food is most commonly served to a consumer in fixed portions, although buffets may be a viable option. Tableware size has been increasing over the past century and more [35, 36]—with the most common sizes of plates and wine glasses sold currently in England similar to the largest tableware used in the current studies [36, 37]. If further studies corroborate that tableware size has an impact on initial servings and this has a subsequent effect on consumption, then regulating tableware size may be an effective intervention and viable policy option in settings where individuals self-serve or self-pour (e.g. buffets or restaurants)—particularly when only one serving is possible [35, 36].

## Conclusions

These two laboratory studies investigated the impact of different sizes and shapes of plates on food selection and different sizes of wine glasses and bottles on alcohol selection. Smaller tableware (i.e. plates and wine glasses) decreased the amount of food and wine self-served in an initial serving. Future studies are required to generate estimates on selection and consumption in real world settings when numerous servings are possible.

## Supplementary Information

**Additional file 1**. Supplementary material.

## Data Availability

The dataset generated and analysed is available on the Open Science Framework project page: https://osf.io/z5msn/ and  the Cambridge University Repository: 10.17863/CAM.75243.

## Abbreviations

ABV: Alcohol by Volume
BMI: Body Mass Index
CI: Confidence Interval
ISRCTN: International Standard Randomised Controlled Trial Number
Ml: Millilitres
OSF: Open Science Framework
SD: Standard deviation
SEP: Socioeconomic Position

## References

[1] Rehm J, Guiraud J, Poulnais R, Shield KD (2018). Alcohol dependence and very high risk level of alcohol consumption: a life-threatening and debilitating disease. Addiction Biology.

[2] Stanaway JD, Afshin A, Gakidou E, Lim SS, Abate D, Abate KH (2018). Global, regional, and national comparative risk assessment of 84 behavioural, environmental and occupational, and metabolic risks or clusters of risks for 195 countries and territories, 1990–2017: a systematic analysis for the Global Burden of Disease Study 2017. The Lancet.

[3] WHO. Obesity and Overweight. Fact sheet. 2018. https://www.who.int/news-room/fact-sheets/detail/obesity-and-overweight. Accessed 24 August 2020.

[4] WHO. Healthy diet. 2020 [cited 2020 Aug 24]. Available from: https://www.who.int/news-room/fact-sheets/detail/healthy-diet.

[5] Hollands GJ, Bignardi G, Johnston M, Kelly MP, Ogilvie D, Petticrew M (2017). The TIPPME intervention typology for changing environments to change behaviour. Nature Human Behaviour.

[6] Robinson E, Nolan S, Tudur-Smith C, Boyland EJ, Harrold JA, Hardman CA (2014). Will smaller plates lead to smaller waists? A systematic review and meta-analysis of the effect that experimental manipulation of dishware size has on energy consumption. Obesity Reviews.

[7] Hollands GJ, Shemilt I, Marteau TM, Jebb SA, Lewis HB, Wei Y (2015). Portion, package or tableware size for changing selection and consumption of food, alcohol and tobacco. Cochrane Database Syst Rev.

[8] Holden SS, Zlatevska N, Dubelaar C. Whether smaller plates reduce consumption depends on who’s serving and who’s looking: a meta-analysis. J Assoc Consum Res. 2016;1(1):134–46.

[9] Munafò MR, Hollands GJ, Marteau TM. Open science prevents mindless science. BMJ. 2018;363:k4309 10.1136/bmj.k4309 PMC6193470 30322848

[10] van der Zee T. The Wansink Dossier: an overview. THE SKEPTICAL SCIENTIST. 2017 [cited 2020 Sep 23]. Available from: http://www.timvanderzee.com/the-wansink-dossier-an-overview/.

[11] Kosīte D, König LM, De-loyde K, Lee I, Pechey E, Clarke N (2019). Plate size and food consumption: a pre-registered experimental study in a general population sample. Int J Behav Nutri Phys Act.

[12] Almiron-Roig E, Forde CG, Hollands GJ, Vargas MÁ, Brunstrom JM (2020). A review of evidence supporting current strategies, challenges, and opportunities to reduce portion sizes. Nutr Rev..

[13] McClain AD, van den Bos W, Matheson D, Desai M, McClure SM, Robinson TN (2014). Visual illusions and plate design: the effects of plate rim widths and rim coloring on perceived food portion size. Int J Obes.

[14] Pilling M, Clarke N, Pechey R, Hollands GJ, Marteau TM (2020). The effect of wine glass size on volume of wine sold: a mega-analysis of studies in bars and restaurants. Addiction.

[15] White AM, Kraus CL, McCracken LA, Swartzwelder HS (2003). Do college students drink more than they think? Use of a free-pour paradigm to determine how college students define standard drinks. Alcohol Clin Exp Res..

[16] Attwood AS, Scott-Samuel NE, Stothart G, Munafò MR (2012). Glass Shape Influences Consumption Rate for Alcoholic Beverages. PLOS ONE.

[17] Langfield T, Pechey R, Pilling M, Marteau TM (2018). Impact of glass shape on time taken to drink a soft drink: A laboratory-based experiment. PLOS ONE.

[18] Langfield T, Pechey R, Gilchrist PT, Pilling M, Marteau TM (2020). Glass shape influences drinking behaviours in three laboratory experiments. Sci Rep..

[19] Stewart PC, Goss E (2013). Plate shape and colour interact to influence taste and quality judgments. Flavour.

[20] Hannan-Jones M, Capra S (2018). Impact of type, size and shape of plates on hospital patients’ perceptions of the quality of meals and satisfaction with foodservices. Appetite.

[21] Codling S, Mantzari E, Sexton O, Fuller G, Pechey R, Hollands GJ (2020). Impact of bottle size on in-home consumption of wine: a randomized controlled cross-over trial. Addiction.

[22] Koh J, Pilner PT (2009). The effects of degree of acquaintance, plate size, and sharing on food intake. Appetite..

[23] Kersbergen I, Oldham M, Jones A, Field M, Angus C, Robinson E (2018). Reducing the standard serving size of alcoholic beverages prompts reductions in alcohol consumption. Addiction.

[24] Sealed Envelope | Internet randomisation (randomization). Available from: https://www.sealedenvelope.com/randomisation/internet/. Accessed 28 October 2020.

[25] Robinson E, Almiron-Roig E, Rutters F, de Graaf C, Forde CG, Tudur Smith C (2014). A systematic review and meta-analysis examining the effect of eating rate on energy intake and hunger. Am J Clin Nutri.

[26] Fox J (2002). Effect displays in R for generalised linear models. J Stat Softw..

[27] DiSantis KI, Birch LL, Davey A, Serrano EL, Zhang J, Bruton Y (2013). Plate size and children’s appetite: effects of larger dishware on self-served portions and intake. Pediatrics.

[28] Rolls BJ, Roe LS, Halverson KH, Meengs JS (2007). Using a smaller plate did not reduce energy intake at meals. Appetite.

[29] Zlatevska N, Dubelaar C, Holden SS (2014). Sizing up the effect of portion size on consumption: A meta-analytic review. J Mark..

[30] Hollands GJ, Marteau TM, Fletcher PC (2016). Non-conscious processes in changing health-related behaviour: a conceptual analysis and framework. Health Psychology Review..

[31] Marteau TM, Fletcher PC, Hollands GJ, Munafò MM. Changing behavior by changing environments. In: The handbook of behavior change Hagger MS, Cameron LD, Hamilton K, Hankonen N, Lintunen T (Eds). New York, NY: Cambridge University Press.

[32] Pechey R, Attwood AS, Couturier DL, Munafò MR, Scott-Samuel NE (2015). Does Glass Size and Shape Influence Judgements of the Volume of Wine?. PLOS ONE.

[33] Geier AB, Rozin P, Doros G (2006). Unit Bias: A New Heuristic That Helps Explain the Effect of Portion Size on Food Intake. Psychol Sci.

[34] Robinson E, Proctor M, Oldham M, Masic U (2016). The effect of heightened awareness of observation on consumption of a multi-item laboratory test meal in females. Physiol Behav.

[35] Marteau TM, Hollands GJ, Shemilt I, Jebb SA (2015). Downsizing: policy options to reduce portion sizes to help tackle obesity. BMJ..

[36] Zupan Z, Evans A, Couturier DL, Marteau TM (2017). Wine glass size in England from 1700 to 2017: a measure of our time. BMJ.

[37] Nisbets. Plate Size Buying Guide | How to choose crockery: size of plates | Nisbets buying guides. 2020. Available from: https://www.nisbets.co.uk/size-of-plates. Accessed 14 Oct 2020.

