# Supplementary material for: Size and shape of plates and size of wine glasses and bottles: impact on self-serving of food and alcohol
Source: BMC Psychol. 2021 Oct 20;9:163. doi: 10.1186/s40359-021-00645-z (PMC8527815; doi:10.1186/s40359-021-00645-z)
Supplement: Supplementary file 1 — Additional file 1. Supplementary material. [file 40359_2021_645_MOESM1_ESM.docx]

**Size and shape of plates and size of wine glasses and bottles: impact on selection of food and alcohol**

**Supplementary material**

**Natasha Clarke*^a (ORCID:^** **^0000-0003-2375-4510)^, Emily Pechey ^a(ORCID: 0000-0002-2288-2652)^, Rachel Pechey^b (ORCID:^** **^0000-0002-6558-388X)^, Minna Ventsel ^a (ORCID: 0000-0001-8308-9140)^, Eleni Mantzari ^a (ORCID: 0000-0003-3147-5079)^, Katie De-loyde^c (ORCID: 0000-0002-8672-9710)^, Mark A Pilling^a(ORCID: 0000-0002-7446-6597)^, Richard W Morris^d (ORCID: 0000-0002-7446-6597)^, Theresa M Marteau ^a (ORCID:^** **^0000-0003-3025-1129)^, Gareth J Hollands ^a (ORCID: 0000-0002-0492-3924)^**

**Author for correspondence: Natasha Clarke,* [*ncc42@medschl.cam.ac.uk*](mailto:ncc42@medschl.cam.ac.uk)

1. Behaviour and Health Research Unit, Department of Public Health and Primary Care, University of Cambridge, Cambridge, UK
2. Nuffield Department of Primary Care Health Sciences, University of Oxford, UK
3. Tobacco and Alcohol Research Group, School of Psychological Science, University of Bristol, Bristol, UK
4. Bristol Medical School, University of Bristol, Bristol, UK

**S1. Randomisation procedure – difference from protocol**

In the protocol it was specified that the order of randomisation would be concealed from researchers until after consent had been taken, but because there were 12 intervention conditions per participant and this took considerable time to set up, the researcher was made aware of the order of conditions before consent was taken in order to collect data efficiently. However, all participants who attended the study consented to participate and entered the study in the order they were assigned by the random sequence.

**S2. Further analyses**

***Sensitivity analysis 1***

A sensitivity analysis of the primary analysis for both studies was repeated, excluding those participants that guessed the true nature of the study during the debrief questions (n = 72 [51%] participants guessed the true nature of the study and 1 participant had missing data for this variable, therefore there were 67 participants included in sensitivity analysis 1 for both studies). Results are presented in Tables S1a and b.

### Table S1a. Primary analysis data – Study 1 (n = 67)

| **Plate size and shape** | | **Grams of food self-served** | t statistic (df) | *p* |
| --- | --- | --- | --- | --- |
|  |  | Estimated MD (95% CI) |  |  |
| Plate size | Small | -74.81 (-87.24, -62.37) | t (66) = -12.01 | < 0.001 |
|  | Medium | -36.30 (-47.07, -25.54) | t (66) = -6.73 | < 0.001 |
|  | Large | - | - | - |
| Plate shape | Square | -1.05 (-6.56, 4.45) | t (66) = -0.382 | 0.704 |
|  | Round | - | - | - |

MD (Mean difference). CI (confidence interval). df (degrees of freedom).

### Table S1b. Primary analysis data – Study 2 (n = 67)

| **Bottle and glass shape** | | **Millilitres of wine self-served** | t statistic (df) | *p* |
| --- | --- | --- | --- | --- |
|  |  | Estimated MD (95% CI) |  |  |
| Glass size | Small | -35.65 (-42.29, -29.02) | t (66) = -10.728 | < 0.001 |
|  | Medium | -20.32 (-25.49, -15.16) | t (66) = -7.851 | < 0.001 |
|  | Large | - | - | - |
| Bottle size | Small | 3.13 (-1.19, 7.45) | t (66) = 1.446 | 0.153 |
|  | Large | - | - | - |

MD (Mean difference). CI (confidence interval). df (degrees of freedom).

***Sensitivity analysis 2***

A sensitivity analysis of the primary analysis for both studies was repeated, excluding those participants that were deemed by the researcher to not follow the instructions correctly. For example, they served the food as a side dish rather than a main meal, or they specifically said they tried to serve the same amount of food and/or wine each time, e.g. by using the same number of serving spoonfuls) (n = 28 [20%], therefore there were 112 participants included in sensitivity analysis 2 for both studies). Results are presented in Tables S2a and b.

### Table S2a. Primary analysis data – Study 1 (n = 112)

| **Plate size and shape** | | **Grams of food self-served** | t statistic (df) | *p* |
| --- | --- | --- | --- | --- |
|  |  | Estimated MD (95% CI) |  |  |
| Plate size | Small | -84.48 (-96.43, -72.54) | t (111) = -14.013 | < 0.001 |
|  | Medium | -43.32 (-52.54, -34.12) | t (111) = -9.316 | < 0.001 |
|  | Large | - | - | - |
| Plate shape | Square | -1.70 (-3.06, 6.45) | t (111) = -0.707 | 0.481 |
|  | Round | - | - | - |

MD (Mean difference). CI (confidence interval). df (degrees of freedom).

### Table S2b. Primary analysis data – Study 2 (n = 112)

| **Bottle and glass shape** | | **Millilitres of wine self-served** | t statistic (df) | *p* |
| --- | --- | --- | --- | --- |
|  |  | Estimated MD (95% CI) |  |  |
| Glass size | Small | -34.93 (-40.93, -28.93) | t (111) = -11.537 | < 0.001 |
|  | Medium | -18.48 (-23.20, -13.76) | t (111) = -7.761 | < 0.001 |
|  | Large | - | - | - |
| Bottle size | Small | 2.85 (-0.18, 5.88) | t (111) = 1.865 | 0.065 |
|  | Large | - | - | - |

MD (Mean difference). CI (confidence interval). df (degrees of freedom).

***Sensitivity analysis 3***

The primary analysis for Study 1 (plate) was repeated, excluding participant ID 98, which was identified as an outlier (9 standard deviations higher than the mean). Results are presented in Table S3.

### Table S3. Primary analysis data - Study 1 (n = 139) – without outlier

| **Plate size and shape** | | **Grams of food self-served** | t statistic (df) | *p* |
| --- | --- | --- | --- | --- |
|  |  | Estimated MD (95% CI) |  |  |
| Plate size | Small | -72.80 (-82.53, -63.07) | t (138) = -14.799 | < 0.001 |
|  | Medium | -36.30 (-42.95, -29.65) | t (138) = -10.791 | < 0.001 |
|  | Large | - | - | - |
| Plate shape | Square | -3.24 (-0.53, 7.01) | t (138) = -1.699 | 0.092 |
|  | Round | - | - | - |

MD (Mean difference). CI (confidence interval). df (degrees of freedom)

***Further analysis 1***

Study order (the order in which the studies [study 1 and study 2] took place) was added as a covariate to the primary analysis models. Results are presented in Tables S4a and b.

### Table S4a. Primary analysis data (with order of studies as a covariate) – intervention 1 (n = 140)

| **Plate size and shape** | | **Grams of food self-served** | | t statistic (df) | *p* |
| --- | --- | --- | --- | --- | --- |
|  |  | Estimated mean (SE) | Estimated MD (95% CI) |  |  |
| Plate size | Small | 123.52 (3.99) | -76.45 (-86.66, -66.24) | t (139) = -14.806 | < 0.001 |
|  | Medium | 159.44 (5.23) | -40.52 (-48.16, -32.89) | t (139) = -10.491 | < 0.001 |
|  | Large | 199.97 (6.81) | - | - | - |
| Plate shape | Square | 160.21 (4.96) | -1.53 (-5.58, 2.52) | t (139) = -0.748 | 0.456 |
|  | Round | 161.74 (5.11) | - | - | - |
| Order of studies | Study 1 first | 158.67 (6.43) | -4.61 (-20.28, 11.07) | t (139) = -0.581 | 0.562 |
|  | Study 2 first | 163.28 (6.21) | - | - | - |

MD (Mean difference). SE (Standard error). CI (confidence interval). df (degrees of freedom).

### Table S4b. Primary analysis data (with order of studies as a covariate) – intervention 2 (n = 140)

| **Bottle and glass size** | | **ML of wine self-served** | | t statistic (df) | *p* |
| --- | --- | --- | --- | --- | --- |
|  |  | Estimated mean (SE) | Estimated MD (95% CI) |  |  |
| Glass size | Small | 126.47 (3.83) | -33.93 (-39.29, -28.57) | t (139) = -12.518 | < 0.001 |
|  | Medium | 143.06 (4.37) | -17.34 (-21.55, -13.13) | t (139) = -8.139 | < 0.001 |
|  | Large | 160.40 (5.23) | - | - | - |
| Bottle size | Small | 144.25 (4.36) | 1.87 (-1.00, 4.75) | t (139) = 1.287 | 0.200 |
|  | Large | 142.37 (4.44) | - | - | - |
| Order of studies | Study 1 first | 140.03 (5.78) | -6.55 (-21.07, 7.97) | t (139) = -0.892 | 0.374 |
|  | Study 2 first | 146.59 (5.58) | - | - | - |

MD (Mean difference). SE (Standard error). CI (confidence interval). df (degrees of freedom).

***Further analysis 2***

The order in which self-servings occurred (the numbers of times each plate or glass appeared in each position in the order [1^st^, 2^nd^,…, 6^th^]) was added as a covariate to the primary analysis models. Results are presented in Tables S5a and b.

### Table S5a. Primary analysis data (with the numbers of times each plate appeared in each position in the order added as a covariate) – intervention 1 (n = 140)

| **Plate size and shape** | | **Grams of food self-served** | | t / f statistic (df) | *p* |
| --- | --- | --- | --- | --- | --- |
|  |  | Estimated mean (SE) | Estimated MD (95% CI) |  |  |
| Plate size | Small | 123.59 (3.99) | -76.70 (-86.93, -66.47) | t (139) = -14.818 | < 0.001 |
|  | Medium | 159.49 (5.23) | -40.80 (-48.44, -33.15) | t (139) = -10.550 | < 0.001 |
|  | Large | 200.29 (6.80) | - | - | - |
| Plate shape | Square | 160.47 (4.94) | -1.30 (-5.81, 2.52) | t (139) = -0.624 | 0.533 |
|  | Round | 161.77 (5.11) | - | - | - |
| Position in the order (1^st^, 2^nd^,…,6^th^). | | | | F (5, 139) = -0.581 | 0.804* |

* umbrella P value across all 6 comparisons

MD (Mean difference). SE (Standard error). CI (confidence interval). df (degrees of freedom).

### Table S5b. Primary analysis data (with the numbers of times each glass appeared in each position in the order added as a covariate) – intervention 1 (n = 140)

| **Bottle and glass size** | | **ML of wine self-served** | | t / f statistic (df) | *p* |
| --- | --- | --- | --- | --- | --- |
|  |  | Estimated mean (SE)* | Estimated MD (95% CI)* |  |  |
| Glass size | Small | 126.70 (3.81) | -33.95 (-39.31, -28.58) | t (139) = -12.502 | < 0.001 |
|  | Medium | 143.30 (4.35) | -17.35 (-21.58, -13.13) | t (139) = -8.128 | < 0.001 |
|  | Large | 160.65 (5.22) | - | - | - |
| Bottle size | Small | 144.48 (4.35) | 1.87 (-1.03, 4.76) | t (139) = 1.275 | 0.204 |
|  | Large | 142.62 (4.42) | - | - | - |
| Position in the order (1^st^, 2^nd^,…, 6^th^). | | | | F (5, 139) = -0.892 | 0.999* |

* umbrella P value across all 6 comparisons

MD (Mean difference). SE (Standard error). CI (confidence interval). df (degrees of freedom).
